# Paper-based Photocatalysts Immobilization without Coffee Ring Effect for Photocatalytic Water Purification

**DOI:** 10.3390/mi11030244

**Published:** 2020-02-26

**Authors:** Qingwei Li, Huichao Lin, Xiaowen Huang, Maocui Lyu, Hongxia Zhang, Xiaoning Zhang, Ruiming Wang

**Affiliations:** Department of Bioengineering, State Key Laboratory of Biobased Material and Green Papermaking, Advanced Research Institute for Multidisciplinary Science, School of Food Science and Engineering, Qilu University of Technology (Shandong Academy of Sciences), Jinan 250353, China; liqingwei@qlu.edu.cn (Q.L.); linhuichao2020@gmail.com (H.L.); lvmaocui@163.com (M.L.); ruiming3k@163.com (R.W.)

**Keywords:** photocatalysis, microreactor, optofluidics, photocatalytic water purification, paper

## Abstract

Photocatalytic water purification is important for the degradation of organic pollutants, attracting intensive interests. Photocatalysts are preferred to be immobilized on a substrate in order to reduce the laborious separation and recycling steps. To get uniform irradiation, the photocatalysts are preferred to be even/uniform on the substrate without aggregation. Generally, the “coffee ring effect” occurs on the substrate during solvent evaporation, unfortunately resulting in the aggregation of the photocatalysts. This aggregation inevitably blocks the exposure of active sites, reactant exchange, and light absorption. Here, we reported a paper-based photocatalyst immobilization method to solve the “coffee ring” problem. We also used a “drop reactor” to achieve good photocatalytic efficiency with the advantages of large surface area, short diffusion lengths, simple operation, and uniform light absorption. Compared with the coffee ring type, the paper-based method showed higher water purification efficiency, indicating its potential application value in the future.

## 1. Introduction

Water pollution is a serious problem especially in modern industrial cities, which is a bad by-product of the prosperity of industry. Photocatalytic water purification is important for the degradation of organic pollutants [1,2,3,4,5], attracting intensive interest and the development of various research works on photoreactors, photocatalysts, and illumination. However, the photocatalytic efficiency of the traditional slurry method is still disappointing due to the low surface to volume ratio (reported as <600 m^2^·m^−3^), long diffusion length, and low photon utilization [6]. To improve the photocatalytic efficiency, photoreactors should possess the advantages of fast mass transfer and photon transfer.

Optofluidic reactors have intrinsic merits, such as fine flow control, large surface-to-volume ratio, and direct delivery of light to the reaction surface, making them natural photoreactors for photocatalysis [7,8,9,10]. Some studies showed microfluidic methods for achieving good degradation efficiency of polluted water [11,12,13,14]. Zhang et al. reported a simple and efficient microfluidic chip with wedge-structure channel for methylene blue (MB) degradation under UV light irradiation [15]. Wang et al. demonstrated a microfluidic photoelectrocatalytic reactor for water purification, in which the positive and negative bias potentials were applied to suppress the electron/hole recombination [4]. Madeddine Azzouz et al. reported a rapid and effective photocatalysis microreactor which integrates in situ grown ZnO nanowires (NW) as an efficient photocatalytic nanomaterial layer. This ZnO NW layer provided an enhanced surface area, minimizing the required interaction time to produce efficient purification performance [16]. Li et al. proposed an optofluidic microreactor with staggered micropillars in the reaction microchamber to mainly enlarge the surface area for loading catalysts and increase the active surface area [17]. In these works, the photocatalysts were all immobilized in different ways because photocatalysts are preferred to be immobilized on a substrate in order to reduce the laborious separation and recycling steps [18].

In our previous work [19], we developed a drop reactor method, which showed the merits of large surface areas, short diffusion lengths, simple operation, as well as higher efficiency than the traditional slurry method. However, a serious “coffee ring effect” occurred during the evaporation process, unfortunately resulting in the aggregation of the photocatalysts, which could inevitably block the exposure of active sites, reactant exchange, and light absorption. Thus, making the photocatalysts even/uniform on the substrate without aggregation (coffee ring effect) in a drop reactor has significant potential applications. 

In this work, we reported a paper-based photocatalyst immobilization method to solve the “coffee ring” problem. A mesoporous graphite carbon nitride (mpg-C_3_N_4_) was chosen to be the photocatalyst, because it has been proven to be a good photocatalyst material in both others’ works and our previous work [9,20,21,22,23,24,25]. Experiments on photo-degradation of methylene blue were adopted to test the effect of both coffee ring and paper-based methods. With the same concentration of mpg-C_3_N_4_, the degradation rate of the paper-based method is much higher/faster than the coffee ring method. The color of methylene blue in the paper-based method showed obvious lightening after 6 min, which can be clearly distinguished by the naked eye. Thus, this simple and highly efficient method with the advantages of being “coffee ring effect-free” and uniform light absorption offers a convenient tool for both photocatalytic water purification and self-cleaning paper development. 

## 2. Materials and Methods 

### 2.1. Drop-reactor Methods: The Coffee Ring Method and Paper-based Method 

**Figure 1:**
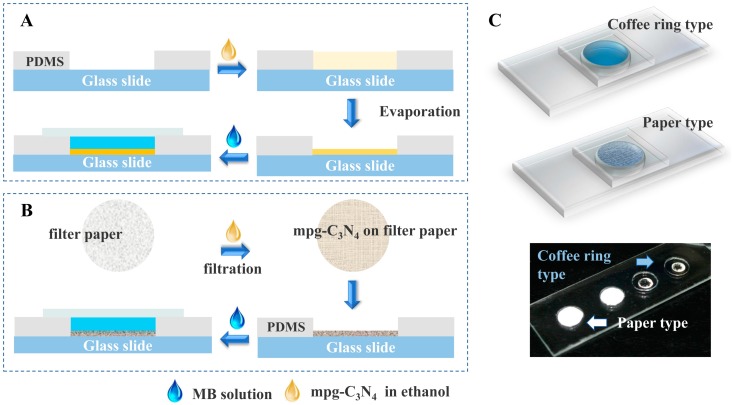
(**A**) Diagram of the coffee ring type drop reactor method, which occurs on the substrate during solvent evaporation, unfortunately resulting in the aggregation of the photocatalysts. (**B**) Diagram of the paper based drop reactor method. During the filtration process, photocatalysts were trapped evenly on the filter paper. (**C**) The schematic and photo of the coffee ring method and paper based method, clearly showing the inevitable aggregation of the photocatalysts in the coffee ring method.

The drop-reactor method is followed as conducted in our previous paper [19]. The uncross-linked polydimethylsiloxane (PDMS, 10:1) was first poured onto a glass slide. After the PDMS layer was cured, we punched wells with a thickness of ~0.35 mm and a diameter of 9 mm. The schematic illustration of the process is shown in Figure 1A,B. Mpg-C_3_N_4_ in ethanol (1 mg/mL, 1× and 2 mg/mL, 2×) were dispersed by ultrasound for 30 min, respectively. 

Fabrication of the coffee ring (CR) type reactor (Figure 1A): 20 μL of the mpg-C_3_N_4_ suspension was added to the prepare hole. The ethanol was removed by evaporation at 50 °C. The left part of the reactor is mpg-C_3_N_4_ (20 μg or 40 μg, equal to CR-1X or CR-2X, a radius of 2.5 mm), which shows an obvious coffee ring effect. After adding the reaction solution (20 μL), a thin glass slide was placed on the well to avoid evaporation. 

Fabrication of paper-based reactor (Figure 1B): the mpg-C_3_N_4_ suspension was filtered by a piece of qualitative medium-speed filter paper, and the concentration (g/m^2^) was adjusted to the concentration used in the coffee ring type reactor. In detail, the concentration of mpg-C_3_N_4_ on the filter paper was calculated by the amount of mpg-C_3_N_4_ in ethanol over the area of the filter paper (g/m^2^). Based on the natural function of the qualitative medium-speed filter paper (high porosity, small pore size), almost all mpg-C_3_N_4_ dispersed in the liquid could be trapped in the process of vacuum filtration. During the evaporation of ethanol, no aggregation of the materials occurs, thus resulting in uniform distribution. The reaction solution (20 μL) was dropped on the mpg-C_3_N_4_-paper and covered with a thin glass slide.

### 2.2. Synthesis of mpg-C_3_N_4_

The mpg-C_3_N_4_ was synthesized as follows [26]: 5 g of cyanamide and 12.5 g of Ludox-HS 40 colloidal silica suspension (1:1 of solid ratio) were mixed until the cyanamide was completely dissolved. An oil bath at 100 °C was used to remove water. After grinding the obtained white solid with a mortar and pestle, we transferred it to a crucible and heated it with the lid on under air at 2.3 °C min^−1^ up to 550 °C (4 hours) and kept the sample at 550 °C for another 4 hours. The resultant yellow powder was treated with 4 M NH_4_HF_2_ solution and stirred for 48 hours. The dispersion was then filtered; the precipitate was thoroughly rinsed with deionized water and ethanol. After the filtering procedure, the yellow powder was dried under vacuum at 60 °C overnight. In this work, we used the same batch of mpg-C_3_N_4_ as in our former work [19], thus the characterization of mpg-C_3_N_4_ is the same as the former data. 

### 2.3. Degradation of MB

A methylene blue (MB) solution of a concentration of 3 × 10^−4^ mol^−1^ was used as the model chemical for characterizing the photoreactivity. Degradation of MB was carried out using a Xenon lamp (300 W, PerfectLight, Beijing, China) through a 420-nm cut-off filter.

## 3. Results

### 3.1. Drop-reactor Methods: The Coffee Ring Method and Paper-based Method 

As shown in Figure 1A, the photocatalysts were suspended in ethanol first and then deposited on the glass substrate during solvent evaporation. Unfortunately, this also resulted in the coffee ring effect, which means the aggregation of the photocatalysts occurred on the substrate. However, the paper based method benefited from the filtration process, in which the photocatalysts were trapped evenly on the filter paper during negative pressure filtration process (Figure 1B). In Figure 1C, the schematic and photo clearly show the differences between the two methods. To clearly understand the morphology and distribution of the photocatalysts on the filter paper, we characterized the sample by the SEM as shown in Figure S1. 

### 3.2. Photocatalytic Degradation of MB

**Figure 2:**
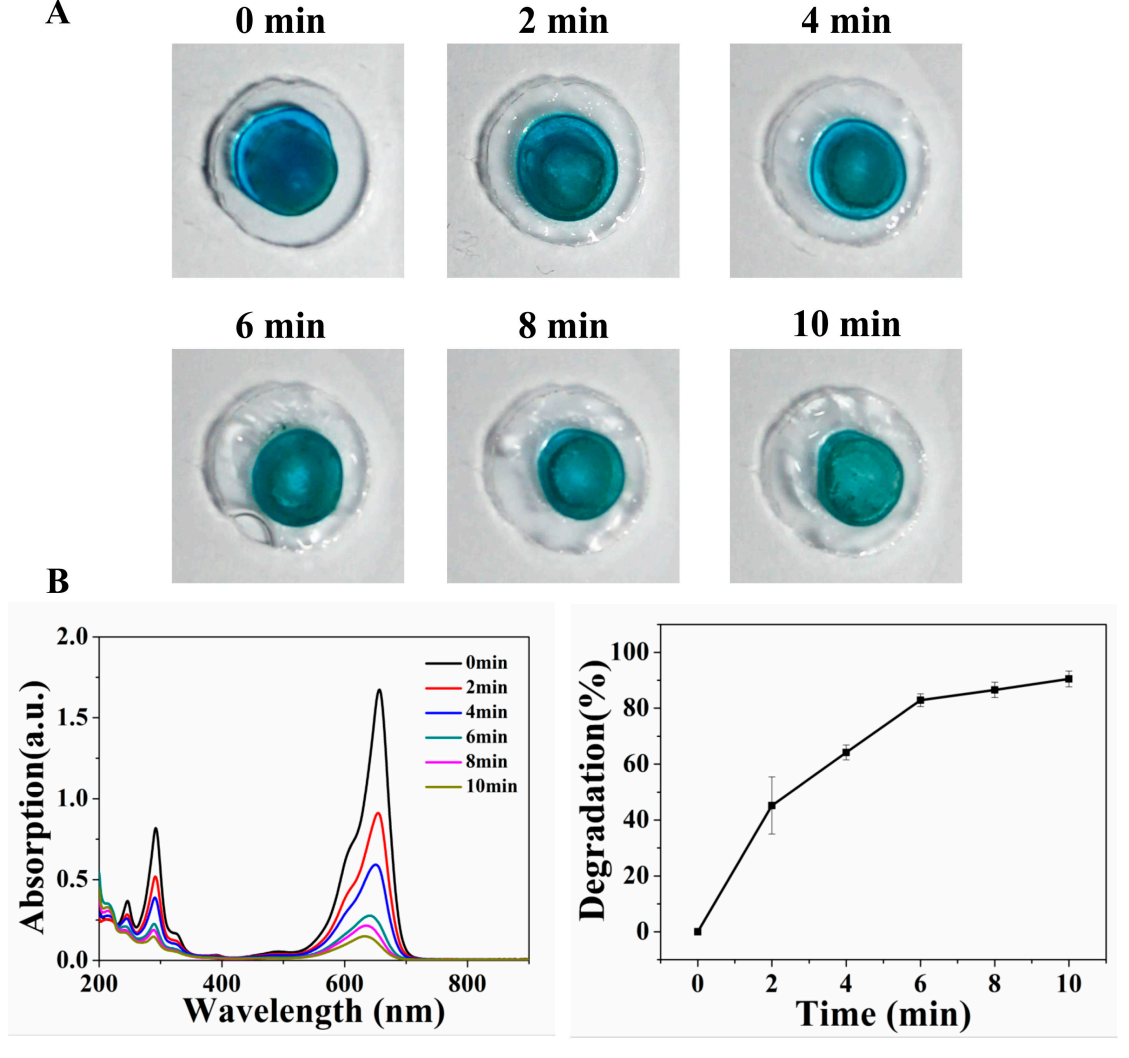
Degradation results of the coffee ring drop reactor method. (**A**) The blue color became gradually lighter as illumination time increased (0–10 min). (**B**) UV-vis spectra of the MB solution after illumination, and the corresponding degradation rates. Each test was repeated three times.

The prepared coffee ring method drop reactor and paper based drop reactor were used to perform the photocatalytic degradation of MB. Figure 2 shows the degradation results of the coffee ring method (CR-2×). The blue color became gradually lighter over the increase in illumination time (Figure 2A), and the UV-vis spectra (Figure 2B) correspondingly presents a clear decreasing trend. Based on the typical peak (656.5 nm) of MB, the standard curve is shown in Figure S2. The degradation rate (%) was calculated by (C_0_-C*_t_*)/C_0_. C_0_ is the concentration of MB at 0 min, C*_t_* is the concentration of MB at *t* min.

**Figure 3:**
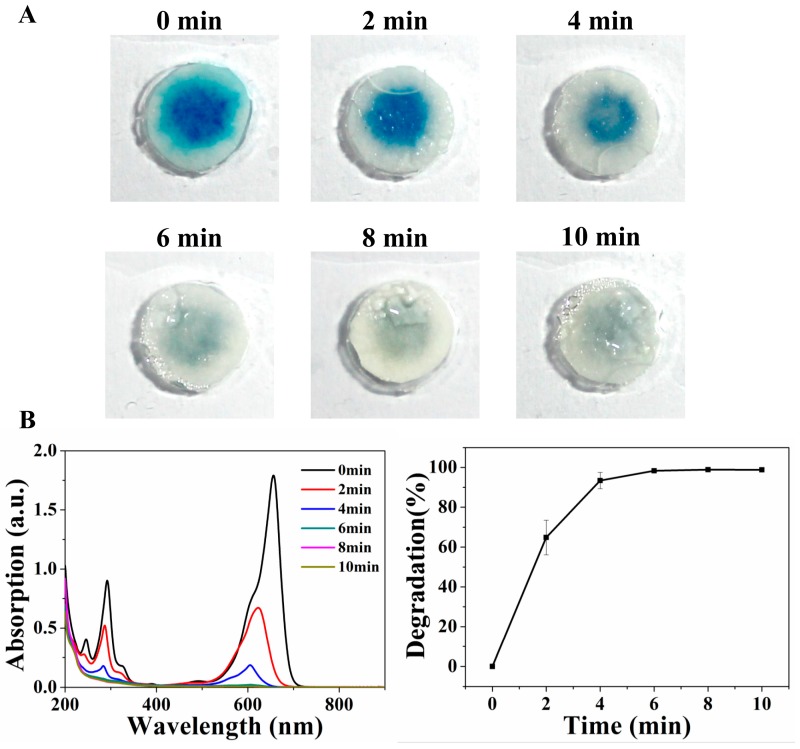
Degradation results of the paper based method. (**A**) The blue color obviously changed after illumination (0–10 min). (**B**) The corresponding UV-vis spectra and the degradation rate. Each test was repeated three times.

Figure 3 shows the degradation results of the paper based drop reactor method (2×). The blue color became obviously lighter after illumination (Figure 3A) and the corresponding UV-vis spectra (Figure 3B) present a clear decreasing trend. Compared with the coffee ring method, the paper based method showed much higher degradation results at the same time point. For example, at 4 min, the degradation rate of the coffee ring method was 64% while the degradation rate was 94% in the paper based method, and at 10 min, the degradation rater was 85% in the coffee ring method while ~100% in the paper based method. 

**Figure 4:**
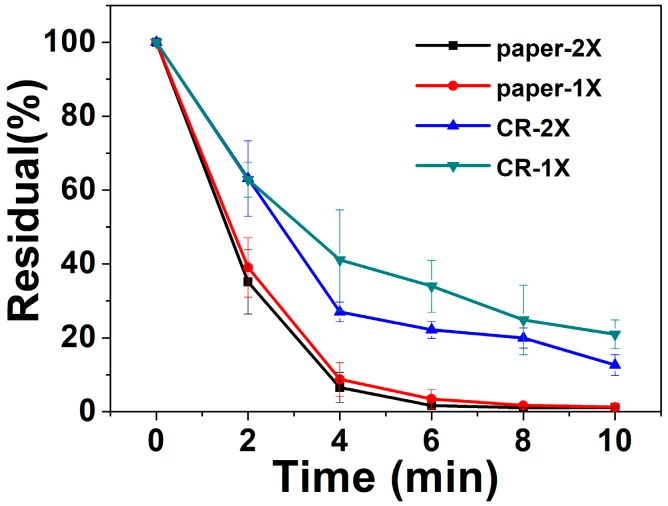
Comparison of the two methods with different loading amounts of mpg-C_3_N_4_. Degradation results of Paper type (paper-1× and paper-2×) show better efficiency than the coffee ring method (CR-1× and CR-2×). Paper-2× shows similar results to paper-1×, and CR-2× only shows a slightly better efficiency than CR-1×, corresponding to the aggregation of photocatalysts in the coffee ring method, which is an obvious reason for the slow reaction rate.

To further explain the possible reason for the slow reaction rate in the coffee ring method, different amounts of photocatalysts were tested and the results were shown in Figure 4. The residual rate (%) was calculated by C*_t_*/C_0_. With different amounts of photocatalysts (1× or 2×), the paper based method showed better degradation ability than the coffee ring method. CR-2× showed a slightly better efficiency than CR-1×, corresponding to the aggregation of photocatalysts in the coffee ring method, which is an obvious reason for the slow reaction rate. Paper-2× showed similar results as paper-1×, indicating the saturation of the illumination site or reaction site. To get a better understanding of the reaction rate, the residual was plotted as a function of time with the exponential relationship:

*y* = *A·*exp(−*x*/*t_c_*) + *y*_0_ (1)

where *t_c_* is the characteristic chemical time, defined as the time required for the concentration of *A* to fall from its initial value to a value equal to 1/e times of the initial value. 

After fitting the data in the origin, we found the degradation rate of paper-1× was 1.69 times of CR-1×. CR-2× is only 1.11 times of CR-1×. 

## 4. Discussion

In this work, the paper-immobilized photocatalysts method shows better degradation efficiency than the coffee ring method. This improvement can be attributed to the lack of aggregation and the uniform irradiation features, which cause the better exposure of active sites, reactant exchange, and light absorption. 

## 5. Conclusions

We proposed a paper-based photocatalyst immobilization method for photocatalytic water purification, which showed better degradation efficiency than the evaporation method (due to coffee ring effect formation). The paper-based immobilization method, with the advantages of high efficiency, no aggregation, and uniform light absorption, offers a convenient tool for both photocatalytic water purification and self-cleaning paper development in the future.

## Supplementary Materials

The following are available online at https://www.mdpi.com/2072-666X/11/3/244/s1, Figure S1: To clearly understand the morphology and distribution of the photocatalysts on the filter paper, we characterized the sample by the SEM, Figure S2: Standard curve of MB solution, showing the relationship between concentration and absorption.
