# Supplementary material for: Paper-based Photocatalysts Immobilization without Coffee Ring Effect for Photocatalytic Water Purification
Source: Micromachines (Basel). 2020 Feb 26;11(3):244. doi: 10.3390/mi11030244 (PMC7143163; doi:10.3390/mi11030244)
Supplement: Supplementary file 1 [file micromachines-11-00244-s001.pdf]

micromachines

Communication

Supplementary information: Paper-based photocatalysts immobilization without coffee ring effect for photocatalytic water purification

Qingwei Li, Huichao lin, Xiaowen Huang, Maocui Lyu, Hongxia Zhang, Xiaoning Zhang, and Ruiming Wang

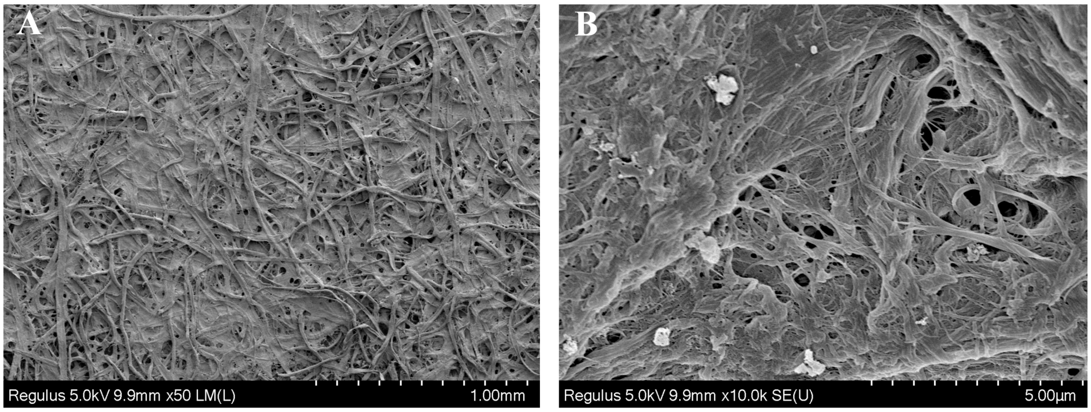

Figure S1. To clearly understand the morphology and distribution of the photocatalysts on the filter paper, we characterized the sample by the SEM. (**A**) Filter paper (**B**) After sonication and filtration, mpg-C_3N_4 trapped/immobilized on the paper (mainly trapped at the hole-like structure).

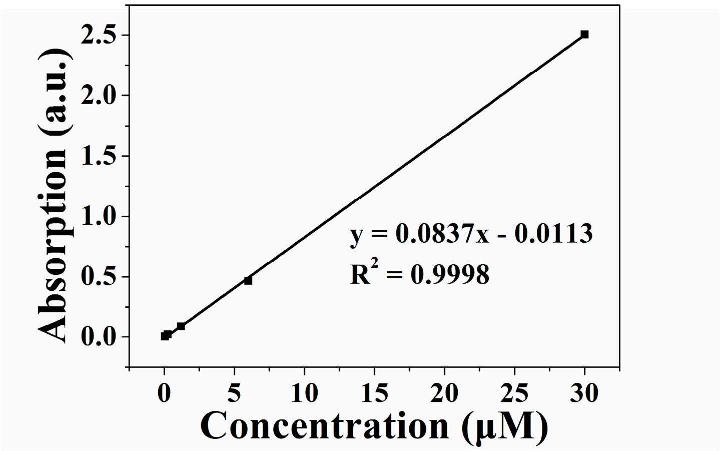

Figure S2. Standard curve of MB solution, showing the relationship between concentration and absorption.
